# A Promising Approach to Effectively Reduce Cramp Susceptibility in Human Muscles: A Randomized, Controlled Clinical Trial

**DOI:** 10.1371/journal.pone.0094910

**Published:** 2014-04-11

**Authors:** Michael Behringer, Markus Moser, Molly McCourt, Johannes Montag, Joachim Mester

**Affiliations:** Institute of Training Science and Sport Informatics, German Sport University Cologne, Cologne, Germany; Tokyo Institute of Technology, Japan

## Abstract

**Background:**

To investigate if the cramp threshold frequency (CTF) can be altered by electrical muscle stimulation in a shortened position.

**Methods:**

A total of 15 healthy male sport students were randomly allocated to an intervention (IG, n = 10) and a non-treatment control group (CG, n = 5). Calf muscles of both legs in the IG were stimulated equally twice a week over 6 weeks. The protocol was 3×5 s on, 10 s off, 150 µs impulse width, 30 Hz above the individual CTF, and was at 85% of the maximal tolerated stimulation energy. One leg was stimulated in a shortened position, inducing muscle cramps (CT), while the opposite leg was fixated in a neutral position at the ankle, hindering muscle cramps (nCT). CTF tests were performed prior to the first and 96 h after the 6^th^ (3 w) and 12^th^ (6 w) training session.

**Results:**

After 3 w, the CTF had significantly (p<0.001) increased in CT calves from 23.3±5.7 Hz to 33.3±6.9 Hz, while it remained unchanged in nCT (pre: 23.6±5.7 Hz, mid: 22.3±3.5 Hz) and in both legs of the CG (pre: 21.8±3.2 Hz, mid: 22.0±2.7 Hz). Only CT saw further insignificant increases in the CTF. The applied stimulation energy (mA^2^ • µs) positively correlated with the effect on the CTF (r = 0.92; p<0.001).

**Conclusions:**

The present study may be useful for developing new non-pharmacological strategies to reduce cramp susceptibility.

**Trial Registry:**

German Clinical Trials Register DRKS00005312

## Introduction

Muscle cramps, defined as sudden, involuntary contractions of muscles that last a few seconds or longer [1], can occur as a consequence of congenital abnormalities or acquired medical diseases [2]. However, there is also a high overall yearly incidence (37%) of muscle cramps in the general population [3]. Nocturnal cramps and cramps during the day time can substantially reduce the quality of life [4], [5], [6]. Further, exercise induced muscle cramps frequently impede an athlete's ability to train or even compete. Besides the hamstrings, the calf muscles (M. gastrocnemius and M. soleus) rank among the most common affected muscle groups [7], [8], [9], [10]. To relieve acute muscle cramps, the affected muscles are usually stretched instinctively by afflicted subjects. Though this can be effective in most cases, on some occasions the muscle cramp returns after stretching, before finally subsiding. Apart from this acute-treatment, there is a lack of evidence regarding the long-term management or prevention of muscle cramps. Blyton et. al [5], who recently published a Cochrane review on that issue, came to the conclusion that there is an overwhelming lack of evidence for non-drug therapies for lower limb muscle cramps. Despite a broad range of available therapy approaches (e.g. physical exercise, weight loss, stretching, massage, heat therapy, compression garments, night ankle dorsiflexion splints), the review identified only one randomized trial which assessed the effectiveness of a non-drug treatment on lower limb muscle cramps. This trial, though impaired by serious limitations in design, assessed the effectiveness of daytime calf muscle stretching in preventing nighttime muscle cramps [11]. After publication of the Cochrane review, one additional randomized controlled trial [12] published supporting evidence of the effectiveness of calf and hamstring stretching before sleep on nocturnal leg cramps. This approach was the subject of debate in several editor's correspondences published by the Journal of the American Medical Association [13], [14], [15], [16]. In part, the insufficient evidence on effective therapy approaches might be explained by the incomplete information available on the cause.

Though the exact pathophysiological mechanism underlying the phenomenon of muscle cramps still remains unclear, different theories on this topic have been proposed and are extensively reviewed elsewhere [1]. According to one of the most recent theories in that field of research, increased afferent muscle spindle activity is accompanied by decreased inhibitory activity of the Golgi tendon organ (GTO), resulting in abnormal alpha motoneuron activity [17]. The latter is characterized by firing frequencies comparable to those observed during voluntary contractions. The neuromuscular theory is supported by the fact that muscle cramps almost exclusively occur at short muscle lengths and are relieved by muscle stretch [18]. In the shortened position it appears that the inhibitory effect of the GTO seems to be largely depressed, while the excitation threshold of motor-endplates are contemporaneously decreased [17].

It is well known that cramp susceptibility varies largely among healthy individuals. While some seem to be almost resistant against muscle cramps, others suffer regularly from these involuntary and painful contractions. Interestingly, it has been previously shown that cramp susceptibility is correlated with an individual cramp threshold frequency (CTF), defined as the minimum electrical stimulation frequency required to elicit a muscle cramp [19]. Miller et al. [19] reported a significant (p<0.001) lower CTF in subjects with a positive cramp history (14.9±1.3 Hz) than in those with a negative (25.5±1.6 Hz). According to previously published literature, the CTF can be increased by muscle fatigue [20] or by ice bag applications [21] and the duration of electrically induced muscle cramps can be reduced by pickle juice ingestion [22]. However, these findings are limited to acute effects, measured immediately after the respective treatment. To the best knowledge of the authors, no investigation to date has presented an effective treatment that increases the individual CTF beyond these acute alterations.

The primary aim of the present investigation was not to induce CTF changes but to examine if structural (hypertrophy) and functional (strength) adaptations differ between calf muscles stimulated either in a neutral or a shortened position (data in preparation), when stimulated above the individual CTF. This induces muscle cramps only in the shortened muscle, while fixating the ankle in a neutral position hinders the cramp development. CTF measurements were added to the testing procedures, to assess if this threshold is somehow affected by the chosen protocol. Even though we did not expect large effects on CTF, we found huge increments in CTF 96 h after the 6^th^ and the 12^th^ training session in those calf muscles that were stimulated in a shortened position. These data are presented here.

## Materials and Methods

The protocol for this trial and supporting CONSORT checklist are available as supporting information; see Checklist S1 and Protocol S1 and S2.

### Ethics Statement

The study has been performed according to the Declaration of Helsinki and the procedures have been approved by the local institutional review board of the German Sport University Cologne. All participants gave their written, informed consent prior to enrollment in the study. The present study was not registered in a clinical trial registry before enrolment of participants started because the intervention was initially planned to be sports- and not health-related (German Clinical Trials Register; Registration number: DRKS00005312).

### Participants

Fifteen healthy male sport students (age 25.2±3.0 years) were recruited from June to August 2013 to take part in a study, which tested a novel (and surely unusual) training method to increase muscle strength and cross sectional area of calf muscles. None of the participants complained about an extraordinary cramp susceptibility defined as one or two muscle cramps per week [7]. Subjects were excluded from the study if they had a history of any neurological conditions, cardiovascular diseases, or if they reported any injuries of the musculoskeletal system during the six months prior to the study. To estimate the percentage of body fat of participants, a bio-impedance analysis was performed using the segmental body composition analyzer BC-418 (Tanita, IL, USA). A summary of subject characteristics can be found in table 1.

**Table 1:** Baseline subject characteristics for each group.

	IG (n = 10)	CG (n = 5)
**Age (years)**	25.9±3.3	23.8±2.4
**Body height (cm)**	179.6±6.1	180.4±5.2
**Body weight (kg)**	78.9±6.2	79.2±8.3
**Body fat (%)**	11±3.6	10.5±2.5
**MVC plantar flexion right (N)**	1389.0±309.0	1452.6±418.9
**MVC plantar flexion left (N)**	1325.0±222.9	1358.2±310.3

n  =  number of participants.

### Trial Design and Randomization

**Figure 1:**
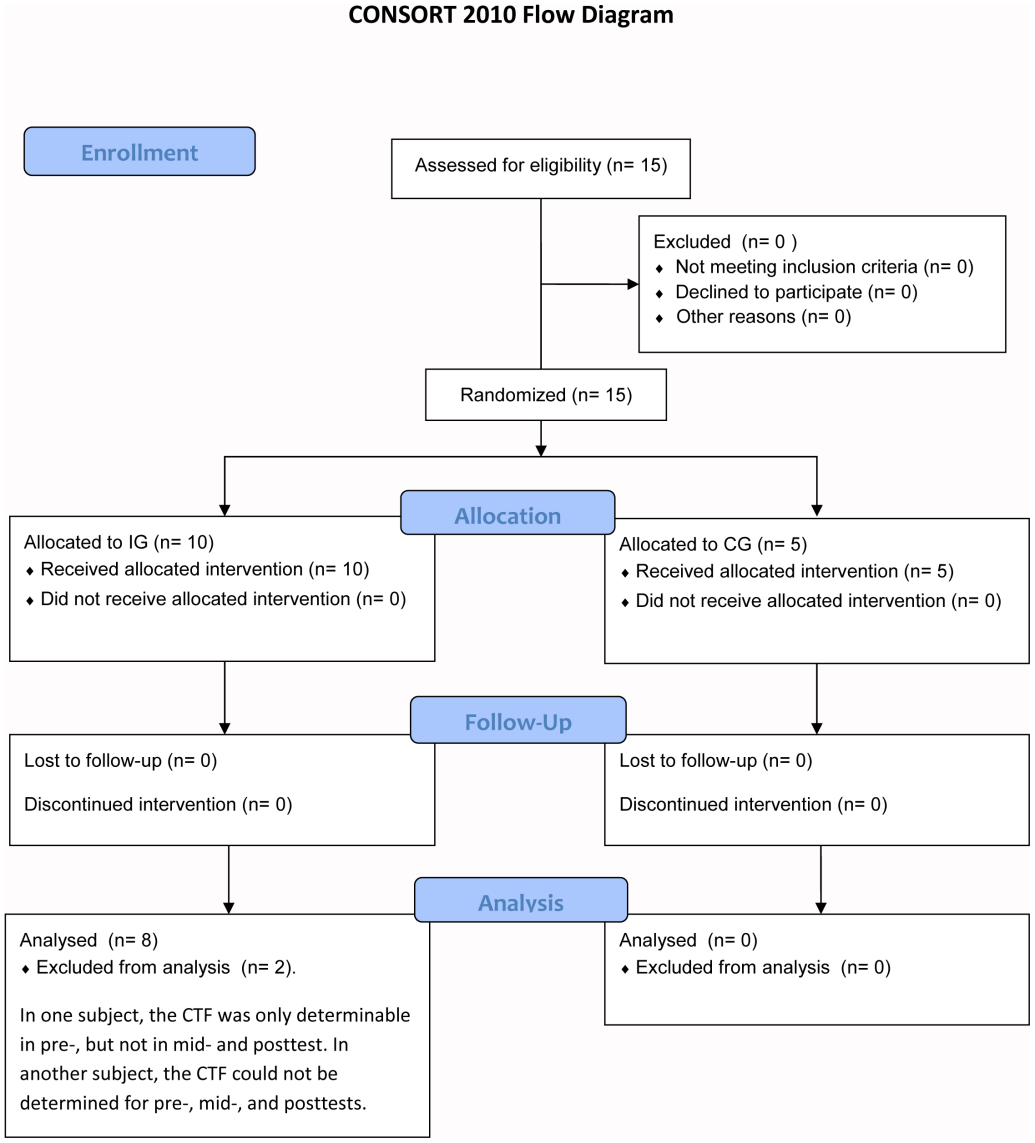
CONSORT flow chart, depicting the passage of participants through the four different stages of the present trial: enrollment, allocation to the intervention (IG) and control group (CG), follow-up, and analysis.

The study was designed as a non-blinded, controlled, incomplete fractional study with imbalanced randomization (2∶1) and took place at the laboratory of the Institute for Training Science and Sport Informatics of the German Sport University Cologne. Participants were randomly assigned to either an intervention (IG; n = 10) or a control group (CG; n = 5) (see Fig. 1). Randomization for group assignment was performed by drawing lots. Afterward, coin-tossing decided which of both legs of the IG subjects, were assigned to either the cramp (CT) or the non-cramp training (nCT) program. Allocation sequence was not concealed. The random allocation sequence was generated by one of the authors (MM). The same author enrolled participants and assigned participants to the interventions. Anthropometric values, and muscle performance (based on the pretest MVC level) did not significantly differ between both groups at baseline. None of the participants suffered from muscle cramps of the calf muscles within the last 6 months prior to the study.

### Interventions

Against the background that high intramuscular tension ranks among the most potent anabolic stimuli [23], we initially designed the present six week cramp training intervention to take advantage of the high tension that coincides with skeletal muscle cramps. Cramps were electrically induced unilaterally in calf muscles (CT) of the IG while sitting on an elevated platform, leaving the legs hanging down freely. The opposite leg (nCT) was stimulated with the same settings (frequency, impulse width, and intensity) in a standing upright position, but was hindered from cramping using a custom-built ankle brace, fixating the ankle in a neutral position. The CG did not receive any muscular stimulation during the intervention period, except those associated with testing procedures.

The protocol, which consisted of three sets of biphasic rectangular-wave pulsed currents at 30 Hz above the individual CTF (see below) and an impulse width of 150 µs, was applied twice a week on the medial and lateral head of the m. gastrocnemius of both legs using a handheld battery-powered myostimulator (Cefar Compex, Compex 3 Professional, Compex Médical SA, Ecublens VD). Each set consisted of 5 s contractions, separated by a 10 s break - resulting in a duty cycle of 0.33. These 5 s contractions induced muscle cramps only in the shortened calf muscles of the CT. That is, a total of three cramps per training session were induced in CT.

To apply the stimulus trains to the muscle, a bipolar stimulation setting was used. Four self-adhesive gel electrodes (Dura-Stick plus, Cefar Compex, Compex Médical SA, Ecublens VD) were placed over of both muscle heads (medial and lateral) – one over the motor point (MP) and one over the proximal part of the muscle belly, just below the popliteal cavity. The MPs, defined as the skin area above the muscle belly at which a minimal current is able to elicit a visible muscle contraction, were localized prior to the training intervention by systematically scanning the skin with a small pen electrode (motor point pen, Cefar Compex, Compex Médical SA, Ecublens VD). This search was performed at low frequency (30 Hz) and low current (2 mA) settings. Only when the MP-search was without results, was the current increased by ∼1 mA steps. The determined MP positions were marked with permanent ink and participants were instructed to refresh this mark daily.

The maximal energy (5,760,000 mA^2^ •µs) of the electrical pulses delivered by the Compex 3 stimulator is defined as the square of the maximal current (120 mA) times the maximum impulse width (400 µs) and each of the 1000 available energy level (EnL) represents 1/1000 of this maximal energy. Increasing the energy level at a selected impulse width of 150 µs is initially (up to 350 EnS) accomplished by increasing the current from 0–120 mA. This is then followed by an increase in impulse width from 150–400 µs until the maximal energy of the device is reached (from 351 to 1000 EnS). The energy of the applied impulses during training protocols was set to 85% of the maximal stimulation energy tolerated (mSET) in the shortened muscle, which was tested prior to each training day following a standardized warm-up protocol of 3×10 calf raises at moderate velocity. The training days were scheduled so that there was two days rest between each training session. During the mSET tests, participants were seated in the same position on the platform as for the CT. Subsequently, the investigator constantly increased the current applied to the calf of the CT leg until participants reported that their mSET was reached. The stimulation was stopped immediately and the reached current intensity was recorded. To ensure that the calves of the cramping and the non-cramping leg perceived the same amount of electrical stimulation during the study period, calf muscles of both legs were stimulated equally (i.e. the current was increased to the mSET value with the same stimulation settings) in an alternating fashion. However, during stimulation of the non-cramping leg, the ankle was fixed in a neutral position and participants were instructed to stand up and to hold an upright position (i.e. hip and knee angle at 180°) in order to avoid the development of a muscle cramp. The rationale for adjusting stimulation intensity to the mSET in the cramping and not the fixed neutral position was that the perceived pain during stimulation in the former position was consistently higher in all subjects tested in a pre-study (unpublished data).

### Outcomes

The individual CTF, defined as the minimal electrical stimulation frequency that elicits a muscle cramp, of the medial head of the m. gastrocnemius was determined in both legs of subjects in the IG and CG as primary endpoint with respect to efficacy in cramp prophylaxis. For that purpose, impulse trains of 5 s were applied to the muscle belly via self-adhesive gel electrodes (Axion GmbH, Germany) using a portable battery-powered myostimulator (Stim-Pro X9, Axion GmbH, Germany). While the impulse width and the current intensity were held constant at 150 µs and 40 mA, respectively, the frequency of stimuli was gradually increased by 2 Hz following 55 s rest periods until a cramp was elicited. If no cramp was induced up to a frequency of 40 Hz, the test was aborted. In addition to the cramp sensation reported by the subject, visual inspection and palpation of the muscle belly by the investigator were used to determine if a cramp was induced at a certain frequency. During the testing procedure, participants lay prone on an examination bench with their ankle joints flexed at 120° and a neutral position in hip and knee joints. The participants were instructed to relax as much as possible to avoid interfering voluntary contractions. CTF tests were performed prior to (pre), midway into (mid), and at the end (post) of the six week intervention. Mid and post tests were performed 96 h after the 6^th^ and the 12^th^ training session, respectively. This deviates from the study protocol as we initially planned to measure the CTF every two weeks. However, in order to reduce time and effort of participants we decided to reduce the measurement frequency. The interim (midway) analysis was performed to get a closer insight into the temporal evolution of CTF changes. It has been previously shown that the applied method reliably measures the individual CTF and that this method is well tolerated in comparison to previously described neurostimulation methods [24].

### Sample Size

Due to the novelty of the applied intervention, no data from previous studies were available to estimate valid inputs for sample size calculation. However, based on the assumption that the induced muscle cramps are associated with extraordinary high intramuscular tension, we expected large effects in terms of muscle hypertrophy (data in preparation), which was the root idea of the present study. To detect a large effect (effect size f = 0.5) with an alpha error probability of 0.05 and a power (1-β error probability) of 0.8, a required sample size of 15 was calculated. These values were generated with the “variance explained by effect” set to 1.0 and the “variance within group” set to 4.0 (SD  = 2.0).

### Statistical Analyses

The differences of CTF changes between the applied protocols and time effects were determined by an analysis of variance (ANOVA) with repeated measurements (three levels) and by post-hoc Bonferroni tests (Statistica for Windows, 7.0, Statsoft, Tulsa, OK). Effect sizes (ESs) were calculated as the difference between the standardized mean change for the treatment and control groups divided by the pooled pretest standard deviation [25]. To compute the statistical power of the group by time interaction, revealed by the performed ANOVA, a post-hoc power analysis using the G*Power software package (version 3.1.4, Franz Faul, Kiel University, Kiel, Germany) was performed. Data presentation used means and standard deviations. The dose-response relationship between mSET and CTF changes was assessed by linear regression analysis, after linearity was verified by residual plot examination. In cases of heteroscedasticity of data, heteroscadicity adjusted standard errors were used, according to Hayes and Cai [26]. The strength of association between mSET and CTF was estimated by calculating the Pearson correlation coefficient. The level of significance was set to <0.05 for all analyses.

## Results

**Figure 2:**
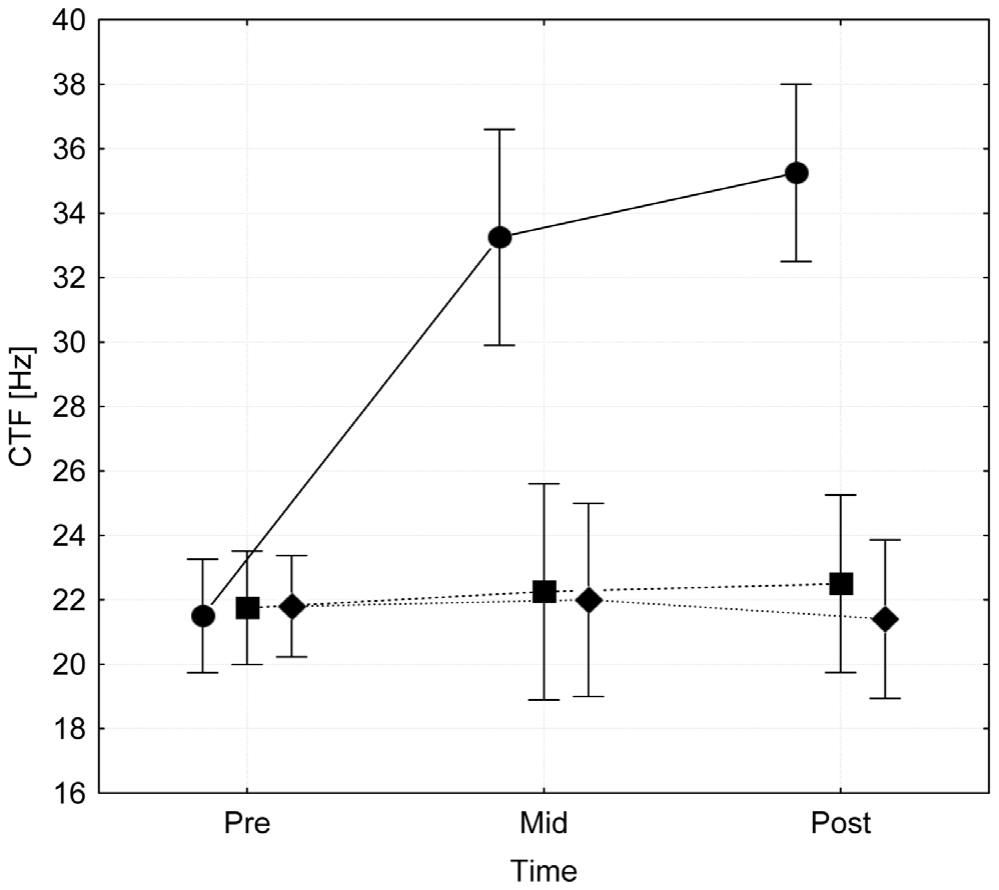
Cramp threshold frequencies (CTF) of calf muscles (m. gastrocnemius medialis) measured at pre-, mid- (3 weeks), and post- (6 weeks) tests following an electrical muscle stimulation protocol that was applied in a shortened (circles) or a neutral position (squares). Further, CTF values of the non-trained legs of the control group (diamonds) are displayed. Vertical bars represent 0.95 confidence intervals. *  =  sign. different from pre values (p<0.001), #  =  sign. difference between groups (p<0.001).

The study ended when the length of the 6-week follow-up goal was reached. All of the enrolled subjects completed the entire training period. The attendance rate was 100%, indicating that the applied program was well tolerated. None of the participants was injured due to the intervention or any other reason. After a period of three weeks, the CTF of the calf muscles stimulated in a shortened position (CT) was significantly increased from 23.3±5.7 to 33.3±6.9 (p<0.001) after three weeks and 35.3±6.0 (p<0.001) after six weeks of intervention (Fig. 2). By contrast, no significant improvement could be found for either the opposite leg, which was stimulated in a neutral position but with the same stimulation settings (pre: 23.6±5.7, mid: 22.3±3.5, post: 22.5±2.1), or the calf muscles of both legs in the CG (pre: 21.8±3.2, mid: 22.0±2.7, post: 21.4±2.3) (Fig. 2). The estimated ESs for CTF changes from pre to mid test measurements were 1.82 (95% CI: 0.57–3.08) and −0.28 (95% CI: −1.36–0.80) for CT and nCT, respectively. From pre to post test, the ES for CT was 2.30 (95% CI: 0.95–3.66), while that for nCT was −0.13 (95% CI: −1.20–0.94).

In one subject, the CTF was only determinable in pre-, but not in mid- and posttest. In another subject, the CTF could not be determined for pre-, mid-, and posttests. The estimated power for observed group by time interaction was 1.0.

The mean mSET, and consequently the applied training energy (85% of mSET), increased linearly over the time order of training sessions. However, the scatter plot of standardized residuals on standardized predicted values fanned out in terms of heteroscedasticity. The respective regression equation was EnL  = 72.25+27.59 • x, with x being the number of performed training sessions. Using heteroscedasticity adjusted standard errors the respective p-level was <0.001. That is, the applied EnL could be increased on average by 28 mA^2^ ·µs per training session. The coefficient of correlation between the number of training sessions and EnL was r = 0.44 (p<0.01).For subjects in which the CTF could be determined (n = 8), a dose-response relationship was found between the overall training energy applied (oEnL), calculated as the sum of all energy level applied per session, and CTF changes after six weeks of intervention (ΔCTF). The respective regression equation was: ΔCTF (Hz)  = 25.40 (Hz) +0.01 · oEnL (mA^2^). The coefficient of correlation obtained between oEnL and ΔCTF was significant (r = 0.92, r2 = 0.85, n = 8, p<0.001).

## Discussion

The main finding of the present investigation was that stimulating calf muscles in a shortened position (CT) was able to significantly increase the individual CTF of subjects from 23.3±5.7 to 35.3±6.0 after a period of six weeks. Results after the first half of the intervention period showed that CTF values had already increased significantly only in the calf muscles that were stimulated in the shortened position (CT). It is important to note that either mid- (3 weeks), and post- (6 weeks) tests of CTF were conducted 96 h after the last training session. To the knowledge of the authors, these data are the first of its kind. As denoted introductorily, the few studies available to date reporting CTF increments were limited to acute effects, measured immediately after the respective interventions [20], [21], [22].

The neural mechanisms underlying these observations remain to be determined. However, according to the aforementioned neuromuscular theory, it might be speculated that set-point changes for GTO and/or muscle spindles are responsible for that observation. Usually, both proprioceptors detect deviations from set-points and thereafter influence the effector system (comprising alpha motor-neurons and extrafusal fibers) via Ia and IIb axons to return the system towards the set-points [27]. Against this background, cramp training may have adapted stretch sensors to forceful contractions in the shortened muscle, resulting in an increased inhibitory feedback of the GTO and/or to a decreased muscle spindle activity during contractions at short muscle lengths. This alteration would help prevent the imbalance between the excitatory and inhibitory drive to the alpha-motoneuron from muscle spindles and GTO, respectively, which has been suggested to underlie the development of muscle cramps [28]. Increasing the inhibitory drive of GTOs is also used when trying to relieve an acute muscle cramp by stretching. The increased tendinous tension accompanied with muscle stretch activates the GTO which will, for their part, increase the frequency of inhibitory impulses to the alpha motor neuron [2]. Interestingly, this method has been reported to be effective regardless of the cause of muscle cramps [28]. Therefore, it seems possible that an altered GTO and/or muscle spindle set-point would be comparably effective in preventing muscle cramps. In this context it should be noted that evidence on the effectiveness of stretching to prevent cramps is contradictory. While the study from Hallegraef et al. [12] found a reduced frequency and severity of nocturnal leg cramps in older adults, Coppin and colleagues [11] concluded that stretching failed to improve those items.

Reports on adaptations in terms of altered set-points of muscle tension sensors are sparse. In cat muscles it could be shown that GTOs reliably signal whole muscle active and passive tension, even if a muscle's force production has been disturbed by fatigue or eccentric exercise [29]. This indicates that there is no set-point change following these acute disturbances. By contrast, the only available study on GTO function in humans has shown that the autogenic inhibition of muscle-force production was decreased during moderate contractions [30]. This led to the assumption that sensitivity changes of GTOs are part of the neural adaptations induced by resistance training, which results in a disinhibition and therefore, to an increased force expression [31]. However, responses to acute and long-term influences may differ substantially. In a comprehensive review from Hutton et al. [32] on the acute and chronic adaptations of muscle proprioceptors to an increased use, it was stated that evidence concerning adaptive responses of GTOs to chronic stimuli is non-existent. Unfortunately, at present, over 20 years later, the same statement holds true.

Changes of afferent information reaching the motor neurons, may not be required to alter the cramp susceptibility. As reported previously, persistent inward currents in motor neurons, as they occur during muscle cramping, seem to affect the relation between synaptic input and motor neuron output in favor of the latter [1]. This amplification of afferent input might be dampened by exercise induced adaptations. More precisely, the intra motor neural data processing may be altered as a result of the present cramp training protocol. Alternatively, neurotransmitter release from afferent fibers might be reduced despite an unchanged impulse rate in Ia and Ib axons. Interestingly, analysis of the present data revealed a distinct dose-response relationship between oEnL and ΔCTF in CT legs. That is, the more energy applied to the calf muscles during the intervention, the greater subjects benefit from the stimulation regarding CTF changes. The small sample size at enrollment and the fact that in two subjects no CTF values could be determined at post- or at pre- and posttest, resulted in only eight complete data sets, on which this correlation is based. However, we found a significant difference in spite of the small sample size. Against this background, it can be assumed with reasonable certainty that the applied training energy is, in fact, of central importance to induce the desirable CTF changes. Since increasing applied energy is associated with an increase in perceived discomfort, this may limit the effectiveness of the present investigation and should be considered when balancing the benefits and harms.

Even though the results of the present study are encouraging, it remains to be determined, if the same effects can be induced in subjects that suffer from increased cramp susceptibility. Furthermore, it needs to be clarified if improvements also hold true for situations in which the muscle cramps occur due to pronounced muscle fatigue, dehydration-electrolyte imbalance, or other reasons. The time it takes to induce the desired effects by cramp training and how long the CTF increments will last remains to be determined. The present data may be limited by the fact that the onset of muscle cramps was assessed using only the sensation of subjects and the visual inspection and palpation of the muscle belly. That is, reliability of cramp detection would have possibly been improved by using electromyographic procedures, as described elsewhere [33]. However, internal consistency between pre- and posttest CTF values in CG calf muscles was found to be excellent in the present study (Cronbach alpha 0.95).

## Conclusion

The cramp training-induced reduction of the individual CTF found 96 h after the 6^th^ and after the 12^th^ training session is a novel finding. No other study to date presented a non-pharmacological approach that induced CTF increments of that duration. Therefore, the present results may be useful for developing new non-pharmacological strategies to reduce cramp susceptibility. Existing data on approaches to prevent skeletal muscle cramps concentrated on muscle stretching [11], [12] and presented conflicting results. Further, these studies were based solely on questionnaires and lacked an objective measurement of the cramp susceptibility. Due to the fact that drugs, acting on the central nervous system, are frequently prescribed as a treatment for muscle cramps despite a lack of evidence for their efficacy [1]. The present findings may be a meaningful and cost-effective alternative approach. Even though the present results are encouraging, they should be validated in a larger cohort of men and women. Future work in this field of research should focus on the underlying neuronal mechanisms, the durability of effect, and the minimum amount of training needed to induce comparable results.

## Supporting Information

Checklist S1 **CONSORT Checklist.**
(DOCX)

Protocol S1 **Trial Protocol (German Version).**
(DOCX)

Protocol S2 **Trial Protocol (English Version).**
(DOCX)

## References

[1] Minetto MA, Holobar A, Botter A, Farina D (2013) Origin and development of muscle cramps. Exerc Sport Sci Rev 41: 3–10. 23038243 10.1097/JES.0b013e3182724817

[2] Schwellnus MP, Derman EW, Noakes TD (1997) Aetiology of skeletal muscle ‘cramps’ during exercise: a novel hypothesis. J Sports Sci 15: 277–285. 9232553 10.1080/026404197367281

[3] Jansen PH, Joosten EM, Vingerhoets HM (1990) Muscle cramp: main theories as to aetiology. Eur Arch Psychiatry Neurol Sci 239: 337–342. 2140785 10.1007/BF01735062

[4] Allen RE, Kirby KA (2012) Nocturnal leg cramps. Am Fam Physician 86: 350–355. 22963024

[5] Blyton F, Chuter V, Walter KE, Burns J (2012) Non-drug therapies for lower limb muscle cramps. Cochrane Database Syst Rev 1: CD008496. 22258986 10.1002/14651858.CD008496.pub2 PMC6481449

[6] Hawke F, Chuter V, Burns J (2013) Impact of nocturnal calf cramping on quality of sleep and health-related quality of life. Qual Life Res 22: 1281–1286. 23011494 10.1007/s11136-012-0274-8 PMC11444264

[7] Jansen PH, Joosten EM, Van Dijck J, Verbeek AL, Durian FW (1991) The incidence of muscle cramp. J Neurol Neurosurg Psychiatry 54: 1124–1125. 1783937 10.1136/jnnp.54.12.1124 PMC1014704

[8] Abdulla AJ, Jones PW, Pearce VR (1999) Leg cramps in the elderly: prevalence, drug and disease associations. Int J Clin Pract 53: 494–496. 10692732

[9] Leung AK, Wong BE, Chan PY, Cho HY (1999) Nocturnal leg cramps in children: incidence and clinical characteristics. J Natl Med Assoc 91: 329–332. 10388258 PMC2608508

[10] Miller TM, Layzer RB (2005) Muscle cramps. Muscle Nerve 32: 431–442. 15902691 10.1002/mus.20341

[11] Coppin RJ, Wicke DM, Little PS (2005) Managing nocturnal leg cramps—calf-stretching exercises and cessation of quinine treatment: a factorial randomised controlled trial. Br J Gen Pract 55: 186–191. 15808033 PMC1463088

[12] Hallegraeff JM, van der Schans CP, de Ruiter R, de Greef MH (2012) Stretching before sleep reduces the frequency and severity of nocturnal leg cramps in older adults: a randomised trial. J Physiother 58: 17–22. 22341378 10.1016/S1836-9553(12)70068-1

[13] Daniell HW, Pentrack J (2013) Improved calf stretch for nocturnal cramp prevention. JAMA Intern Med 173: 934–935. 10.1001/jamainternmed.2013.96 23712410

[14] Garrison SR (2012) Calf stretching prophylaxis for nocturnal cramps-reply. Arch Intern Med 172: 970–971. 10.1001/archinternmed.2012.1971 23752894

[15] Hawke F, Burns J (2012) New evidence for stretching for preventing nocturnal cramps. Arch Intern Med 172: 1770–1771. 10.1001/2013.jamainternmed.123 23229945

[16] Daniell HW (2012) Calf stretching prophylaxis for nocturnal cramps. Arch Intern Med 172: : 970; author reply 971. 10.1001/archinternmed.2012.1393 22732758

[17] Bergeron MF (2008) Muscle Cramps during Exercise - Is It Fatigue or Electrolyte Deficit? Current Sports Medicine Reports 7: S50–S55.

[18] Bertolasi L, De Grandis D, Bongiovanni LG, Zanette GP, Gasperini M (1993) The influence of muscular lengthening on cramps. Annals of Neurology 33: 176–180. 8434879 10.1002/ana.410330207

[19] Miller KC, Knight KL (2009) Electrical stimulation cramp threshold frequency correlates well with the occurrence of skeletal muscle cramps. Muscle Nerve 39: 364–368. 19208394 10.1002/mus.21170

[20] Stone MB, Edwards JE, Huxel KC, Cordova ML, Ingersoll CD, et al (2010) Threshold frequency of an electrically induced cramp increases following a repeated, localized fatiguing exercise. J Sports Sci 28: 399–405. 20131142 10.1080/02640410903508854

[21] Kawahara T, Kikuchi N, Stone MB, Brucker JB, Edwards JE (2005) Ice Bag Application Increases Threshold Frequency of Electrically Induced Muscle Cramp. In: Free communications, oral presentations: Non-Therapeutic Issues of Cryotherapy. J Athl Train 40 Suppl 2S–35.

[22] Miller KC, Mack GW, Knight KL, Hopkins JT, Draper DO, et al (2010) Reflex inhibition of electrically induced muscle cramps in hypohydrated humans. Med Sci Sports Exerc 42: 953–961. 19997012 10.1249/MSS.0b013e3181c0647e

[23] West DW, Burd NA, Staples AW, Phillips SM (2010) Human exercise-mediated skeletal muscle hypertrophy is an intrinsic process. Int J Biochem Cell Biol 42: 1371–1375. 20541030 10.1016/j.biocel.2010.05.012

[24] Minetto MA, Botter A, Ravenni R, Merletti R, De Grandis D (2008) Reliability of a novel neurostimulation method to study involuntary muscle phenomena. Muscle Nerve 37: 90–100. 17912751 10.1002/mus.20903

[25] Hedges LV, Olkin I (1985) Statistical Methods for Meta-analysis: Academic Press.

[26] Hayes AF, Cai L (2007) Using heteroskedasticity-consistent standard error estimators in OLS regression: an introduction and software implementation. Behav Res Methods 39: 709–722. 18183883 10.3758/bf03192961

[27] Bear MF, Connors BW, Paradiso MA (2007) Neuroscience: Lippincott Williams & Wilkins.

[28] Miller KC, Stone MS, Huxel KC, Edwards JE (2010) Exercise-associated muscle cramps: causes, treatment, and prevention. Sports Health 2: 279–283. 23015948 10.1177/1941738109357299 PMC3445088

[29] Gregory JE, Brockett CL, Morgan DL, Whitehead NP, Proske U (2002) Effect of eccentric muscle contractions on Golgi tendon organ responses to passive and active tension in the cat. J Physiol 538: 209–218. 11773329 10.1113/jphysiol.2001.012785 PMC2290032

[30] Chalmers G (2002) Do Golgi tendon organs really inhibit muscle activity at high force levels to save muscles from injury, and adapt with strength training? Sports Biomech 1: 239–249. 14658379 10.1080/14763140208522800

[31] Gabriel DA, Kamen G, Frost G (2006) Neural adaptations to resistive exercise: mechanisms and recommendations for training practices. Sports Med 36: 133–149. 16464122 10.2165/00007256-200636020-00004

[32] Hutton RS, Atwater SW (1992) Acute and chronic adaptations of muscle proprioceptors in response to increased use. Sports Med 14: 406–421. 1470793 10.2165/00007256-199214060-00007

[33] Stone MB, Edwards JE, Babington JP, Ingersoll CD, Palmieri RM (2003) Reliability of an electrical method to induce muscle cramp. Muscle Nerve 27: 122–123. 12508307 10.1002/mus.10296

