# Correction: A Promising Approach to Effectively Reduce Cramp Susceptibility in Human Muscles: A Randomized, Controlled Clinical Trial

**DOI:** 10.1371/journal.pone.0114108

**Published:** 2014-11-14

**Authors:** The PLOS ONE Staff

There is an error in the third sentence of the “Methods” subsection of the Abstract. The correct sentence is: The protocol was 6x5 s on, 10 s off, 150 µs impulse width, 30 Hz above the individual CTF, and was at 85% of the maximal tolerated stimulation energy.

There is an error in the first sentence of the “Results” subsection of the Abstract. The correct sentence is: After 3 w, the CTF had significantly (p<0.001) increased in CT calves from 21.5±1.4 Hz to 33.3±6.9 Hz, while it remained unchanged in nCT (pre: 23.6±5.7 Hz, mid: 22.3±3.5 Hz) and in both legs of the CG (pre: 21.8±3.2 Hz, mid: 22.0±2.7 Hz).

There are multiple errors in the last three sentences of second paragraph of the “Interventions” subsection of the Materials and Methods. The correct paragraph is: Each set consisted of 6 x 5 s contractions, separated by a 10 s break - resulting in a duty cycle of 0.33. These 5 s contractions induced muscle cramps only in the shortened calf muscles of the CT. That is, a total of 18 cramps per training session were induced in CT.

There is an error in the fifth sentence of the Results. The correct sentence is: After a period of three weeks, the CTF of the calf muscles stimulated in a shortened position (CT) was significantly increased from 21.5±1.4 Hz to 33.3±6.9 (p<0.001) after three weeks and 35.3±6.0 (p<0.001) after six weeks of intervention (Fig. 2).

**Figure 2:**
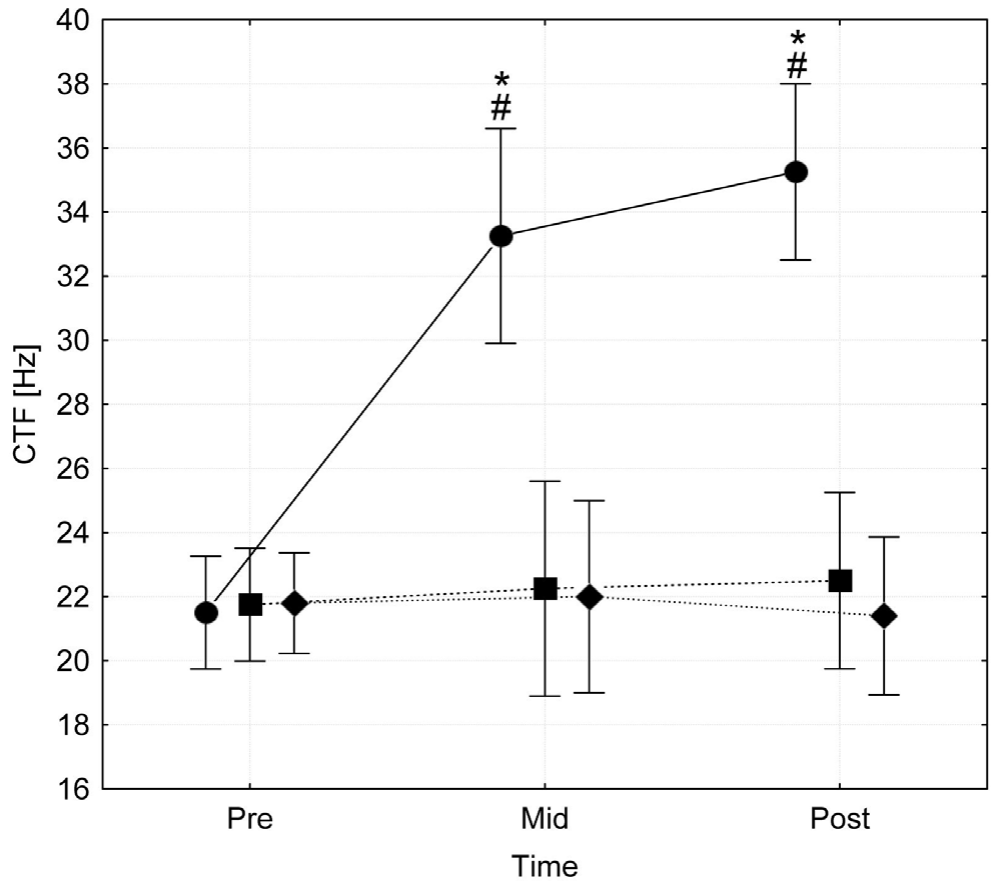
Cramp threshold frequencies (CTF) of calf muscles (m. gastrocnemius medialis) measured at pre-, mid- (3 weeks), and post- (6 weeks) tests following an electrical muscle stimulation protocol that was applied in a shortened (circles) or a neutral position (squares). Further, CTF values of the non-trained legs of the control group (diamonds) are displayed. Vertical bars represent 0.95 confidence intervals. *  =  sign. different from pre values (p<0.001), #  =  sign. difference between groups (p<0.001).

There is an error in the first sentence of the Discussion. The correct sentence is: The main finding of the present investigation was that stimulating calf muscles in a shortened position (CT) was able to significantly increase the individual CTF of subjects from 21.5±1.4 Hz to 35.3±6.0 after a period of six weeks.

There are multiple errors in Figure 2. Please see the corrected Figure 2 here.
