# Supplementary material for: A Promising Approach to Effectively Reduce Cramp Susceptibility in Human Muscles: A Randomized, Controlled Clinical Trial
Source: PLoS One. 2014 Apr 11;9(4):e94910. doi: 10.1371/journal.pone.0094910 (PMC3984281; doi:10.1371/journal.pone.0094910)
Supplement: Protocol S1 — Trial Protocol (German Version). (DOCX) [file pone.0094910.s002.docx]

## Projektbeschreibung

**Zusammenfassung**

Muskelkrämpfe sind plötzlich-auftretende, unfreiwillige und schmerzhafte Kontraktionen eines verkürzten Muskels. Da Krämpfe im Sport leistungslimitierend sein können, befasst sich die Forschung in diesem Kontext mit Maßnahmen zur Reduzierung der Häufigkeit, Dauer und Intensität der Krämpfe. Die niedrigste elektrische Stimulationsfrequenz mit der ein Krampf ausgelöst werden kann, wird als threshold frequency (TF) bezeichnet und hat sich als Parameter der individuellen Krampfneigung etabliert. Zusammenhänge zwischen TF und Tendenz zu Krämpfen, Hydrationszustand oder Ermüdung konnten bereits gezeigt werden. Unklar ist, ob sich die threshold frequency und damit die Krampfneigung durch Training verändern lässt. Ziel dieser Studie ist es den Einfluss einer sechs-wöchigen Trai-ningsintervention mittels elektrisch-induzierter Muskelkontraktionen auf die Krampfnei-gung (TF) sowie auf morphologische und funktionelle Muskelparameter (CSA, MD, MVC) zu untersuchen.

**Stand der Forschung**

Muskelkrämpfe sind ein häufig auftretendes Leiden und stellen eine Beeinträchtigung der Lebensqualität der Betroffenen dar. Definiert werden Krämpfe als plötzlich-auftretende, unfreiwillige und schmerzhafte Kontraktionen eines Muskels, die sich selbst innerhalb von Sekunden oder Minuten wieder auflösen und bei verkürzter Muskulatur auftreten (Minetto et. al, 2013). Die Prävalenz von Muskelkrämpfen liegt zwischen 35% und 50% (Naylor & Young, 1994; Abdulla, Jones & Pearce, 1999) wobei 40% dieser Personen berichten, dass sie öfter als dreimal in der Woche an Krämpfen leiden (Naylor & Young, 1994). Mus-kelkrämpfe treten bei Erkrankungen der Motorneuronen, bestimmten metabolischen Stö-rungen, akuten Veränderungen des Wasser- und Elektrolytgleichgewichts und, ohne ein-deutig diagnostizierbare Gründe, in der Nacht oder bei sportlicher Betätigung auf (Miller, 2005).

Muskelkrämpfe, die während des Sports auftreten, werden als exercise-associated muscle cramps (EAMC) bezeichnet und stellen eine Leistungsbeeinflussung in Wettkampf und Training dar. Der Fokus der Forschung im sportlichen Kontext liegt auf der Aufklärung der Ursachen von EAMCs und den Maßnahmen, die sowohl Prävalenz als auch Dauer und Intensität dieser Muskelphänomene reduzieren. Die maßgeblichen Arbeiten stammen aus den USA (Miller, Stone), Italien (Minetto/Botter) und Südafrika (Schwellnus).

Aufgrund der klinischen Eigenschaften von Muskelkrämpfen (plötzlich-auftretend, unfrei-willig, selbst-auflösend) wurden Wege entwickelt um diese künstlich herbeizuführen. Hier-bei hat sich in den letzten Jahren die elektrische-Stimulation des Nervs (Stone et. al, 2003) oder der Motor-Points (Minetto et. al, 2008) des Muskels zur Krampfinduktion als reliable Methode etabliert. Durch diese electrically-induced muscle cramps (EIMC) wurde ermöglicht, Muskelkrämpfe unter Laborbedingungen zu erforschen. Dabei hat sich die threshold frequency (TF), als gängiger Parameter der Krampfforschung im Sport entwi-ckelt. Die TF ist die niedrigste Frequenz der elektrischen Stimulation, bei der Muskel-krämpfe ausgelöst werden können (Stone et. al, 2003). Miller und Knight (2009) konnten zeigen, dass Personen, die unter Muskelkrämpfen leiden eine niedrigere TF hatten als jene Probanden, ohne Krämpfe in der Vergangenheit. Sie schlossen daraus, dass die threshold frequency ein Maß der individuellen Krampfneigung darstellt. Weiterhin wurde gezeigt, dass Krampfdauer und –Intensität mit Erhöhung der Stimulationsfrequenz über die TF zunehmen womit eine erleichterte Erforschung dieses Muskelphänomens ermög-licht wird (Miller et. al, 2012). Stone et. al (2010) untersuchten, wie sich die lokale Belas-tung eines Muskels auf die threshold frequency auswirkt und konnten zeigen, dass die TF nach Einsatz eines Ermüdungsprotokolls zunimmt. Die gestiegene TF zeigt, dass akute muskuläre Belastung die Krampfneigung senken kann. Es ist jedoch unklar ob sich die threshold frequency langfristig verändern lässt.

Ziel dieser Studie ist es den Einfluss einer sechs-wöchigen Trainingsintervention mittels elektrisch-induzierter Muskelkontraktionen auf die Krampfneigung (TF) sowie auf morpho-logische und funktionelle Muskelparameter zu untersuchen. Als Parameter der Morpholo-gie wird der Muskelquerschnitt („Cross Sectional Area“, CSA) der Wadenmuskulatur er-fasst. Um funktionelle Anpassungen zu ermitteln werden maximale willkürliche Kontrakti-onen („Maximum voluntary contractions“, MVC) durchgeführt. Dabei wird zwischen 1) elektrisch-induzierten Muskelkrämpfen, 2) elektrisch-induzierten Muskelkontraktionen in neutraler Muskellänge und 3) einer Kontrollgruppe unterschieden. Folgenden Hypothesen wird nachgegangen:

1. Die threshold frequency (TF) ist nach Beendigung eines sechs-wöchigen Trainings mittels elektrisch-induzierter Muskelkontraktionen am M. Gastrocnemius höher als vor der Intervention. Die threshold frequency (TF) am Ende der Intervention ver-hält sich wie folgt: TFCramp > TFIso > TFCon
2. Elektrisch-induzierte Muskelkrämpfe resultieren in einer stärkeren Erhöhung der Muskelparameter (CSA, MD, MVC) als die elektrisch-induzierten Kontraktionen in neutraler Muskellänge.

### Versuchsplanung

Bei dieser Studie handelt es sich um eine Trainingsintervention über einen Zeitraum von 6 Wochen mit vorhergehenden, begleitenden und nachfolgenden Datenerhebungen (siehe Abb. 1). Ziel der Studie ist es, Einflüsse des Trainings auf die Krampfneigung (Threshold-Frequency) zu untersuchen sowie chronische Anpassungen des M. gastrocnemius an elektrisch-induzierte Muskelkontraktionen anhand von morphologischen und funktionellen Parametern darzustellen.

**
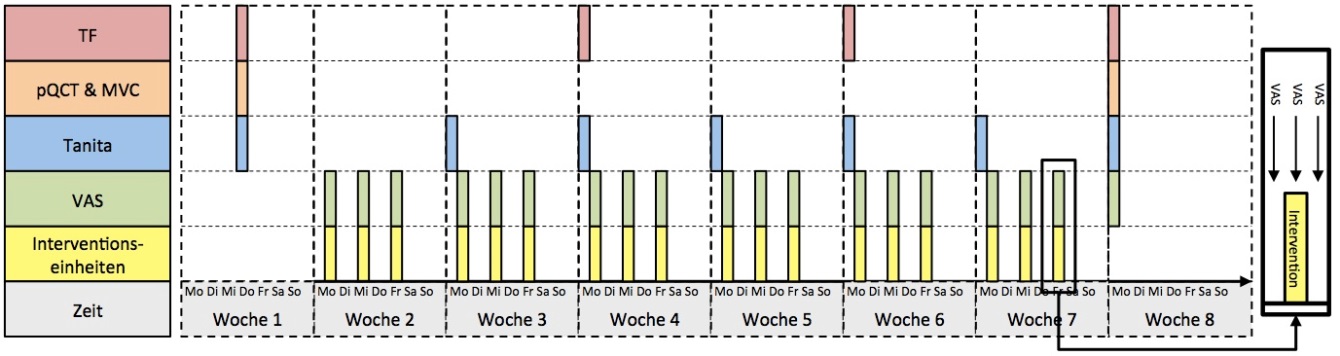
**

**MRT**

**Abb. 1:** Studiendesign – TF: Threshold frequency, MRT: magnetic resonance imaging, VAS: visualized analog pain scale

#### Protokoll

Die Probanden (n=24) werden in eine Interventionsgruppe (n=12) und eine Kontrollgruppe (n=12) eingeteilt. Die Kontrollgruppe durchläuft, wie die Interventionsgruppe, Eingangs- und Ausgangstests, erhält jedoch sonst keine weiteren Interventionen.

Die Intervention findet am M. gastrocnemius über einen Zeitraum von 6 Wochen, wobei wöchentlich dreimal trainiert wird. Die elektrische Muskelstimulation erfolgt mittels Com-pex 3 (DJO, Guildford, England) über zuvor detektierte Motorpoints des M gastrocnemius lateralis und medialis. Jeder Proband der Interventionsgruppe absolviert zwei Interventi-onsarten, die randomisiert am rechten bzw. linken Bein appliziert werden: Der Muskel wird dabei 1) neutral bei einem Sprunggelenkswinkel von 90° oder 2) in verkürzter, maximal plantarflexierter Position stimuliert (siehe Abb. 2b). Die beiden Interventionsarten werden in jeder Trainingseinheit alternierend durchgeführt (siehe Abb. 2a). Dabei werden pro Bein sechs Sätze á sechs Kontraktionen mit einer Satzpause von 90s hervorgerufen. Die Sti-mulationsform ist rechtwinklig mit einem Duty-cycle von 0,33 (5s on, 10s off), einer Fre-quenz von 60Hz und einer Bandbreite von 152μs. Die Impulsamplitude wird über die max. Toleranz zu Beginn der Einheit festgelegt. Die Einstellungen für die Elektromyostimulation sind bei beiden Beinen gleich, lediglich die Position der Probanden und damit die Muskel-länge unterscheiden sich.

Im Anhang (siehe Referenzen) befindet sich eine Tabelle, welche internationale Studien darstellt, die sich der Elektromyostimulation als Methodik bedienen.


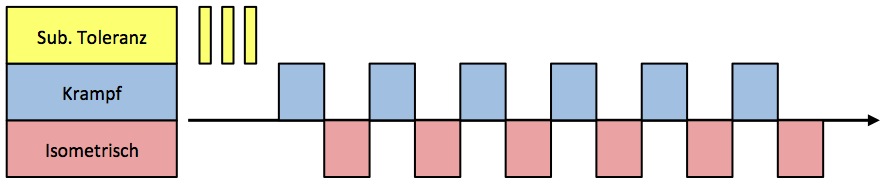

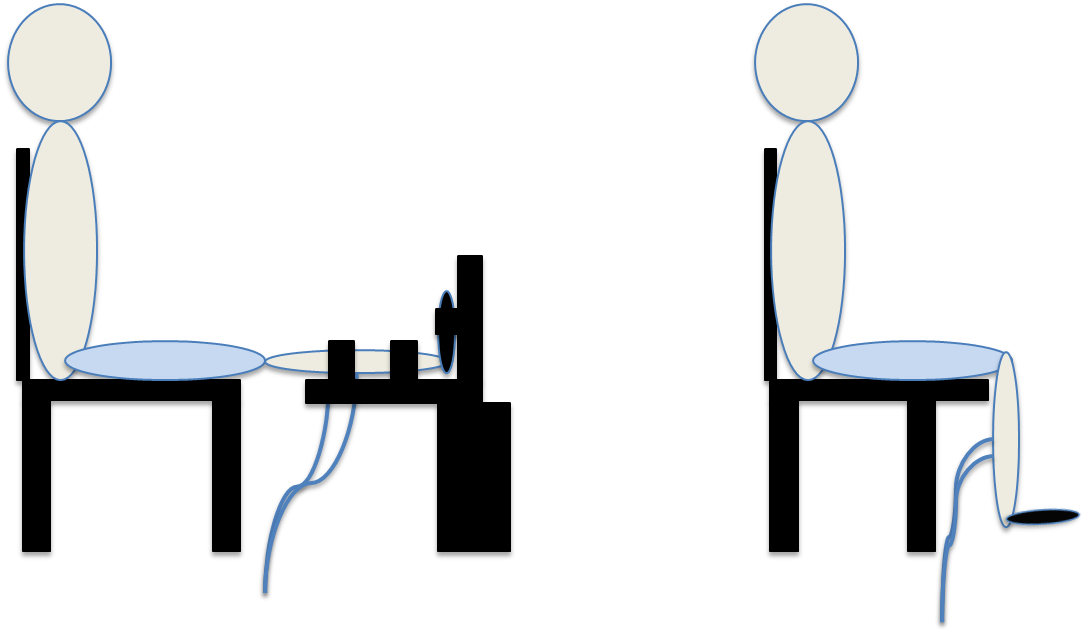


**Abb. 2:** a) Zeitlicher Ablauf einer Interventionseinheit; b) Positionierung der Probanden für die neutrale (links) und die verkürzte Position (rechts).

In Betrachtung des verwendeten EMS-Gerätes, der rechteckigen Impulsform, der Stimula-tionsfrequenz von 60 Hz, der Bandbreite von 152 μs und des angewandten Duty-Cycles von 33% weist unsere Studie große Parallelen zu den oben angeführten Untersuchungen auf. Der gewählte Trainingszeitraum von sechs Wochen und die Trainingshäufigkeit von drei Einheiten pro Woche liegen im Durchschnittsbereich der internationalen Studien.

Die vorhandene Literatur im Bereich der Krampfforschung mit sportwissenschaftlichem Kontext zeigt, dass es während der Studien zu keinen Verletzungen durch die gezielte Auslösung von Krämpfen kam (siehe Tab. 3). Die in unserer Studie durchgeführte Stimu-lationsmethode lehnt sich an die Technik der Forschungsgruppe um Minetto und Botter an.

Da in keiner der oben genannten Studien (siehe Tab. 2 und 3) Schäden auftraten, kann das potenzielle Verletzungsrisiko als sehr gering eingeschätzt werden. Es ist zu erwarten, dass aufgrund der nicht vorhandenen Vorerfahrungen mit EMS und dem Auftreten von Muskelkrämpfen in der ersten Woche ein Muskelkater bei den Probanden auftritt, über den die Beteiligten informiert werden. Da speziell durch EMS von sehr schnellen Adaptio-nen berichtet wird, könnte der Muskelkater bereits in der zweiten Woche ausbleiben.

#### Probandenkollektiv

Voraussetzung für die Teilnahme an der Studie ist ein guter körperlicher und geistiger Gesundheitszustand. Die Teilnehmer sind hauptsächlich männliche Sportstudenten, die in den letzten sechs Monaten keine Verletzungen des muskuloskelettalen Systems der unte-ren Extremität sowie keine kardiopulmonalen Grunderkrankungen vorweisen.

Alle Probanden werden ausführlich über den Untersuchungsablauf, die Untersuchungs-techniken und die möglichen Belastungen aufgeklärt (siehe Probandenaufklärung). So-wohl seitens der Probanden als auch der Studienleitung kann die Untersuchung jederzeit abgebrochen werden.

#### Parameter

Tabelle 1 führt die im Zuge der Studie erhobenen Parameter unter Angabe von Zeitpunk-ten und verwendeten Geräten an.

**Tabelle 1**: Erhobene Parameter

| Parameter | Zeitpunkt | Gerät |
| --- | --- | --- |
| Threshold frequency (TF) | Pre  Post | Compex 3 |
| Maximal voluntary contraction (MVC) | Pre  Post | Beinpresse |
| Muscle cross sectional area (CSA) | Pre  Post | MRT |
| Bio-Impedanz-Analyse (BIA) | Wöchentlich | Tanita |
| Subjektives Schmerzempfinden (VAS) | Jede Einheit | Fragebogen |

#### Referenzen

Abdulla, A. J., Jones, P. W. & Pearce, V. R. (1999). Leg cramps in the elderly: prevalence, drug and disease associations. *International Journal of Clinical Practice, 53*(7), 494–496.

Miller, T. M., & Layzer, R. B. (2005). Muscle cramps. *Muscle Nerve*, *32*(October), 431–442.

Miller, K. C., & Knight, K. L. (2009). Electrical stimulation cramp threshold frequency correlates well with the occurence of skeletal muscle cramps. *Muscle Nerve*, *39*(March), 364–368.

Miller, K. C., Knight, K. L., Wilding, S. R., & Stone, M. B. (2012). Duration of Electrically Induced Muscle Cramp Increased by Increasing Stimulation Frequency. *Journal of Sport Rehabilitation*, *21*, 182–185.

Minetto, Marco Alessandro; Botter, Alberto; Ravenni, Roberta; Merletti, Roberto; De Grandis, D. (2008). Reliability of a novel neurostimulation method to study involuntary muscle phenomena. *Muscle Nerve*, *37*(January), 90–100.

Minetto, M. A., Holobar, A., Botter, A., & Farina, D. (2013). Origin and development of muscle cramps. *Exercise and sport sciences reviews*, *41*(1), 3–10.

Naylor, J. R. & Young, J. B. (1994). A general population survey of rest cramps. *Age and ageing*, *23*(5), 418–420.

Stone, M. B., Edwards, J. E., Babington, J. P., Ingersoll, C. D., & Palmieri, R. M. (2003). Reliability of an electrical method to induce muscle cramp. *Muscle Nerve*, (January), 122–123.

Stone, M. B., Edwards, J. E., Huxel, K. C., Cordova, M. L., Ingersoll, C. D., Babington, J. P., & Carolina, N. (2010). Threshold frequency of an electrically induced cramp increases following a repeated , localized fatiguing exercise. *Journal of Sports Sciences*, *28*(4), 399–405.

#### Studienauflistung zu EMS-Anwendung und Krampfforschung

Die folgenden zwei Tabellen geben einen Überblick der internationalen Studien zu den Bereichen Trainingsanpassungen an Elektromyostimulation und Krampfforschung.

**Tabelle 2:** Darstellung internationaler Studien zu Trainingsanpassungen an Elektromyostimulation

| Autor | Impulsform | Intensität | Impulsbreite [µs] | Frequenz [Hz] | Stimula-tionsdauer [s] | Stimulationspause [s] | Duty-cycle | Verletzungen | Fitnesslevel | Gesamteinheiten | Wochen | Einheiten pro Woche |
| --- | --- | --- | --- | --- | --- | --- | --- | --- | --- | --- | --- | --- |
| Balogun et al. (1993) | monophasischer 2-Peak Nadelimpuls | - | 70 | 20 | 10 | 50 | 16,7 | Nein | untrainiert | 18 | 6 | 3 |
| Balogun et al. (1993) | monophasischer 2-Peak Nadelimpuls | - | 70 | 45 | 10 | 50 | 16,7 | Nein | untrainiert | 18 | 6 | 3 |
| Balogun et al. (1993) | monophasischer 2-Peak Nadelimpuls | - | 70 | 80 | 10 | 50 | 16,7 | Nein | untrainiert | 18 | 6 | 3 |
| Currier und Mann (1983) | "Russian Current"-Sinusimpuls | 66,7% MVC ~ 45,8 mA | 450 | 50 | 15 | 50 | 23,1 | Nein | untrainiert | 15 | 5 | 3 |
| Currier und Mann (1983) | "Russian Current"-Sinusimpuls | 88,4% MVC ~ 55,3 mA | 450 | 50 | 15 | 50 | 23,1 | Nein | untrainiert | 15 | 5 | 3 |
| Eriksson et al. (1981) | Rechtwinkelimpuls | - | 500 | 200 | 15 | 15 | 50 | Nein | trainierte Studenten | 25 | 5 | 5 |
| Eriksson et al. (1981) | Rechtwinkelimpuls | - | 500 | 200 | 6 | 6 | 50 | Nein | trainierte Studenten | 25 | 5 | 5 |
| Fahey et al. (1985) | biphasischer, asymetrischer Rechtwinkelimpuls | 45 mA | - | 50 | 10 | 5 | 66,7 | Nein | untrainiert | 18 | 6 | 3 |
| Gondin et al. (2005) | biphasischer Rechtwinkelimpuls | 68 % MVC ~ 75 mA | 400 | 75 | 6,25 | 20 | 23,8 | Nein | untrainiert | 32 | 8 | 3 |
| Gondin et al. (2005) | biphasischer Rechtwinkelimpuls | 68 % MVC ~ 75 mA | 400 | 75 | 4 | 20 | 16,7 | Nein | untrainiert | 32 | 8 | 4 |
| Herrero et al. (2006) | biphasischer Rechtwinkelimpuls | 40 mA | 400 | 120 | 3 | 30 | 23,8 | Nein | untrainiert | 8 | 4 | 2 |
| Herrero et al. (2006) | biphasischer Rechtwinkelimpuls | 66 mA | 400 | 120 | 3 | 30 | 9,1 | Nein | untrainiert | 16 | 4 | 4 |
| Kubiak et al. (1987) | "Russian Current"-Sinusimpuls | 75% MVC | - | 50 | 15 | 50 | 23,1 | Nein | untrainiert | 15 | 5 | 3 |
| Lai et al. (1988) | biphasischer, asymetrischer Impuls | 64,60% | 200 | 50 | 5 | 5 | 50 | Nein | untrainiert | 15 | 3 | 5 |
| Lai et al. (1988) | biphasischer, asymetrischer Impuls | 52,00% | 200 | 50 | 5 | 5 | 50 | Nein | untrainiert | 15 | 3 | 5 |
| Laughman et al. (1983) | "Russian Current"-Sinusimpuls | 33% MVC ~ 62,5 mA | - | 50 | 15 | 50 | 23,1 | Nein | untrainiert | 25 | 5 | 5 |
| Maffiuletti et al. (2006) | biphasischer Rechtwinkelimpuls | 70% MVC ~ 64 mA | 400 | 75 | 6,25 | 20 |  | Nein | untrainiert | 18 | 4 | 4,5 |
| Maffiuletti et al. (2002) | biphasischer Rechtwinkelimpuls | 60% MVC ~ 60 mA | 400 | 75 | 4 | 20 | 16,7 | Nein | untrainiert | 16 | 4 | 4 |
| Mohr et al. (1985) | monophasischer 2-Peak Nadelimpuls | - | 45 | 50 | 10 | 10 | 50 | Nein | untrainiert | 15 | 3 | 5 |
| Owens und Malone (1983) | "Russian Current"-Sinusimpuls | 60% MVC ~ 46,5 | 200 | 50 | 15 | 50 | 23,1 | Nein | untrainiert | 10 | 1,5 | 6,7 |
| Owens und Malone (1983) | "Russian Current"-Sinusimpuls | 39% MVC ~ 34,6 | 200 | 50 | 15 | 50 | 23,1 | Nein | untrainiert | 10 | 1,5 | 6,7 |
| Romero et al. (1982) | "Faraday"-Impuls (Mittelfrequenz) | - | - | 2000 | 4 | 4 | 50 | Nein | untrainiert | 10 | 5 | 2 |
| Selkowitz (1985) | "Russian Current"-Sinusimpuls | 91% MVC ~ 59 mA | 450 | 50 | 10 | 120 | 7,7 | Nein | trainierte Studenten | 12 | 4 | 3 |
| Stefanovska und Vodovnik (1985) | monophasischer Sinusimpuls | 5% MVC ~ 73,1 mA | 300 | 25 | 10 | 50 | 16,7 | Nein | untrainiert | 24 | 4 | 6 |
| Stefanovska und Vodovnik (1985) | monophasischer Rechtwinkelimpuls | 5% MVC ~ 43,12 mA | 300 | 25 | 10 | 50 | 16,7 | Nein | untrainiert | 24 | 4 | 6 |

**Tabelle 3:** Darstellung internationaler Studien zur Krampfforschung im sportwissenschaftli-chen Kontext

| Autor | Probanden [n] | Muskel | Stimulationsform | Verletzungen |
| --- | --- | --- | --- | --- |
| Minetto et. al (2008) | 19 | M. abductor hallucis | Motor Point | Nein |
| Minetto et. al (2009) | 11 | M. abductor hallucis | Motor-Point | Nein |
| Minetto und Botter (2009) | 11 | M. abductor hallucis  M. flexor hallucis brevis  M. gastrocnemius | Motor Point | Nein |
| Minetto et. al (2009) | 15 | M. abductor hallucis | Motor Point | Nein |
| Bertolasi et. al (1993) | 10 | M. flexor hallucis brevis | Nerv | Nein |
| Braulick et. al (2012) | 9 | M. flexor hallucis brevis | Nerv | Nein |
| Caress et. al (2000) | 14 | M. abductor hallucis | Nerv | Nein |
| Miller und Knight (2007) | 23 | M. flexor hallucis brevis | Nerv | Nein |
| Miller und Knight (2009) | 31 | M. flexor hallucis brevis | Nerv | Nein |
| Miller et. al (2010) | 12 | M. flexor hallucis brevis | Nerv | Nein |
| Miller et. al (2012) | 20 | M. flexor hallucis brevis | Nerv | Nein |
| Miller et. al (2012) | 20 | M. flexor hallucis brevis | Nerv | Nein |
| Serrao et. al (2007) | 13 | M. flexor hallucis brevis | Nerv | Nein |
| Stone et. al (2003) | 16 | M. flexor hallucis brevis | Nerv | Nein |
| Stone et. al (2010) | 16 | M. flexor hallucis brevis | Nerv | Nein |
| Jung et. al (2005) | 13 | M. gastrocnemius | Willkürlich | Nein |
| Khan und Burne (2007) | 13 | M. gastrocnemius | Willkürlich | Nein |
| Roelefeld et. al (2000) | 8 | M. gastrocnemius | Willkürlich | Nein |
