# Supplementary material for: A Promising Approach to Effectively Reduce Cramp Susceptibility in Human Muscles: A Randomized, Controlled Clinical Trial
Source: PLoS One. 2014 Apr 11;9(4):e94910. doi: 10.1371/journal.pone.0094910 (PMC3984281; doi:10.1371/journal.pone.0094910)
Supplement: Protocol S2 — Trial Protocol (English Version). (DOCX) [file pone.0094910.s003.docx]

## Project description

**Summary**

Muscle cramps are sudden, involuntary and painful contractions of shortened muscles. Since cramps can limit sports performance, research focuses on treatments to reduce frequency, duration, and intensity of muscle cramps. The lowest frequency of electrical stimulation that elicits a cramp is denoted as the threshold frequency (TF) and has been established as a parameter of the individual cramp susceptibility. Correlations between TF and cramp susceptibility, hydrational status, or muscle fatigue could already be shown. It remains unclear if the threshold frequency, and therefore the cramp susceptibility, can be altered by training. The aim of the present study is to test the effect of a six-week training intervention, using electrically-induced muscle contractions, on the cramp susceptibility (TF) and on morphologic and functional parameters (CSA, MD, MVC).

**Current state of research**

Muscle cramps are a common ailment and are associated with a significant deterioration in the quality of life of concerned persons. Cramps are defined as sudden, involuntary, painful contractions that dissipate within seconds or minutes and occur in shortened muscles (Minetto et. al 2013). The prevalence of muscle cramps is between 35% and 50% (Naylor & Young, 1994; Abdulla, Jones & Pearce, 1999), and 40% of these subjects report suffering from cramps more than three times a week (Naylo & Young, 1994). Muscle cramps occur in association with diseases of the motor neurons, certain metabolic disorders, acute alterations of the water- and electrolyte homeostasis, and without any diagnosable reasons during sport activities or at night (Miller, 2005).

Muscle cramps that occur during sports are denoted as exercise-associated muscle cramps (EAMC) and affect performance during competition and training. The focus of research within the context of sport lies on treatments to reduce prevalence, duration, and intensity of these muscle phenomenon. Leading research articles come from the USA (Miller, Stone), Italy (Minetto, Botter), and South Africa (Schwellnuss).

Due to clinical characteristics of muscle cramps (sudden, involuntary, self-limiting), techniques have been established to artificially evoke cramps. In this context, the electrical stimulation of the nerve (Stone et. al, 2003) or the motor-points (Minietto et. al, 2008) have been established as a reliable method to evoke cramps. Using these electrically-induced muscle cramps (EIMC), it is now possible to investigate muscle cramps under laboratory settings. In this context, the threshold frequency (TF) has been established as a reliable parameter for cramp research in sports. The TF is defined as the lowest frequency of an electrical stimulation that induces a muscle cramp (Stone et. al, 2003). Miller and Knight (2009) could show that subjects who suffer from muscle cramps have a lower TF than those subjects that have no cramps in their medical history. The authors concluded that the threshold frequency is a measure of the individual cramp susceptibility. Further, it was shown that duration and intensity of muscle cramps increases if the simulation frequency is increased beyond the TF, making the investigation of this muscle phenomenon easier (Miller et al., 2012). Stone et al. (2010), who investigated how loadings of a single muscle affect the threshold frequency, could show that the TF was increased after a fatigue protocol. The increased TF delineates that acute muscular activity is able to reduce the cramp susceptibility. However, it remains unclear, if threshold frequency can be altered over the long term.

The aim of the present study is to assess the effect of a six-week training intervention, consisting of electrically-induced muscle contractions, on the TF and on morphological and functional adaptations of muscle parameters. The cross sectional area (CSA) of calf muscles was used as a morphological parameter. To assess functional adaptations, maximal voluntary contractions (MVC) will be performed. Electrically induced muscle cramps will be induced in one leg (1), while muscles of the opposite leg will be stimulated in a neutral position (2), both legs of a third group of subjects will serve as a control (3). The following hypotheses will be addressed:

1. The threshold frequency (TF) will increase following a six-week training of electrically-induced contractions of the m. gastrocnemius. At the end of the intervention, the threshold frequency will be as follows: TFCramp > TFIso > TFCon
2. Electrically-induced muscle cramps will induce greater adaptations regarding the selected muscle parameters (CSA, MD, MVC) than electrically-induced contractions at a neutral muscle length.

### Study design

The present study is designed as a training intervention over a period of 6 weeks with preceding concomitant and subsequent data assessment (see figure 1.). The aim of the study is to assess the effect of the training, which consists of electrically-induced muscle contractions, on the cramp susceptibility (threshold frequency) and on functional and morphological adaptations of the m. gastrocnemius.

**
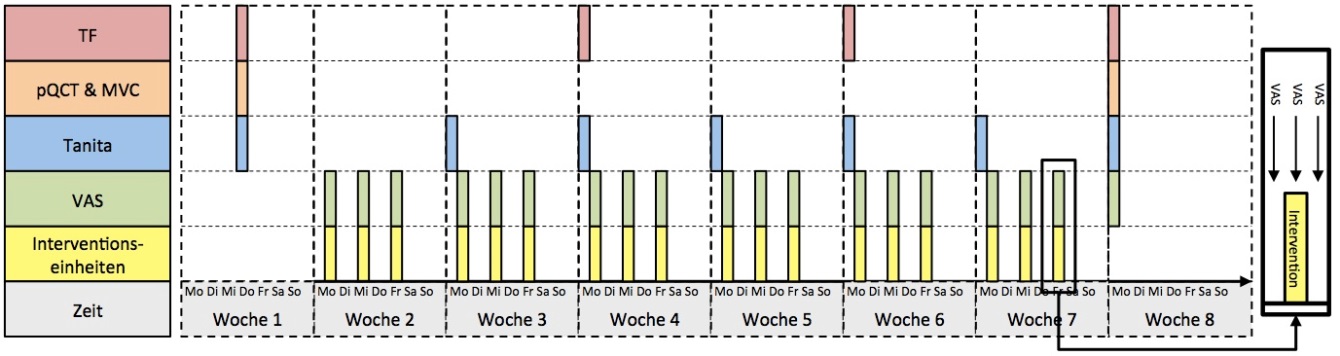
**

**MRT**

**Fig. 1:** Study design - TF: Threshold frequency, MRT: magnetic resonance imaging, Tanita: bioimpedance analysis, VAS: visualized analog pain scale, Interventionseinheiten: training sessions, Zeit: Time, Woche: week.

#### Study protocol

Participants (n = 24) will be allocated to an intervention (n = 12) and a control group (n = 12). The control group will undergo the same pre and posttests as the intervention group, but will receive no other intervention.

The intervention will be conducted on the m. gastrocnemius over a period of 6 weeks with a training frequency of three sessions per week. Electrical stimulation will be performed with Compex 3 (DJO, Guildford, England) over predetermined motor points of the m. gastrocnemius lateralis and medialis. Every participant in the intervention group will perform two different interventions, which will be conducted in a randomized fashion on the right and left leg. The muscle will be either stimulated in a 1) neutral position of the ankle joint of 90° or 2) in shortened, maximally plantarflexed position (see Fig. 2b). Both interventions will be conducted in an alternating fashion during each training (see Fig. 2a). Interrupted by 90s rest intervals, six sets of six contractions will be evoked per leg. The stimulation will consist of rectangular currents of 60Hz frequency, 152µs impulse width, and a duty cycle of 0.33 (5s on, 10s off). The impulse amplitude will be adjusted according to the maximal tolerated current, which will be tested prior to each training session. That is, the applied stimulation protocol will be the same for both legs and only the length of the stimulated muscles will differ.

In the Annex (see References), international studies that used electromyostimulation as a method are listed.


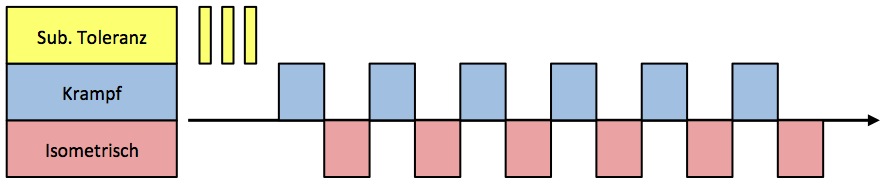

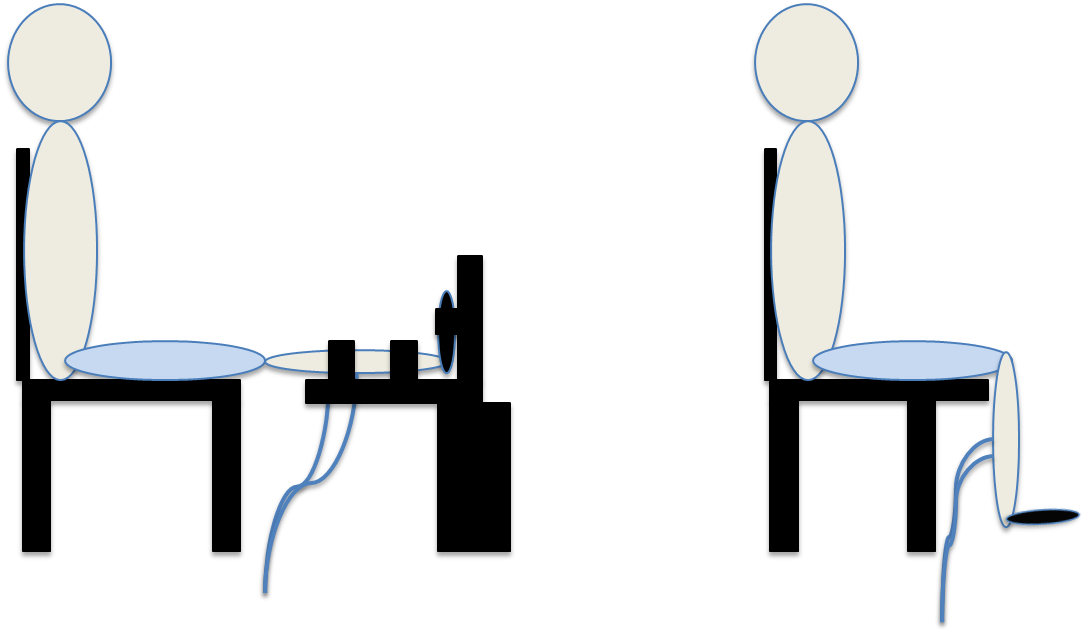


Fig. 2: a) temporal sequence of one training session; b) subject positioning for stimulating the calf muscles in a neutral (left) and in a shortened position (right).

In terms of the used EMS-device, the rectangular pulse form, the stimulation frequency of 60Hz, the impulse width of 152µs, and the duty-cycle of 33%, our study provides numerous parallels to aforementioned studies (see Annex). The chosen training duration of six weeks and the training frequency of three sessions per week are within the average training determinants of previous international studies.

Existing data in the field of muscle cramp research within a sport-scientific context has shown that no injuries occurred due to the artificially provoked muscle cramps (see Tab. 3). The stimulation protocol that will be applied during the planned investigation draws on the technique used by the research group of Minetto and Botter.

Since none of the aforementioned studies reported any damages, the potential injury risk can be rated low. Due to the inexperience of subjects regarding EMS training and the induction of muscle cramps, it is to be expected that subjects will suffer from muscle soreness within the first week. Subjects will be informed about this expectation. Since it has been reported previously that EMS is an associated with a quick adaptation, muscle soreness may already be absent in the second week of intervention.

#### Subjects

A good physical and mental health status are required for taking part at the study. Participants will be male sport students with no injury of the musculoskeletal system of lower limbs within the last six month prior to the study or any cardiovascular diseases.

All participants will be extensively informed about the course of investigation, the applied methods, and potential risks (Handout). The intervention can be stopped at any point by both participants and investigators.

#### Parameter

Table 1 lists all of the parameter that will be measured with the respective measurement time points and the used devices.

**Table 1:** Parameter

| Parameter | Timepoint | Device |
| --- | --- | --- |
| Threshold frequency (TF) | Pre  Post | Compex 3 |
| Maximal voluntary contraction (MVC) | Pre  Post | Leg press |
| Muscle cross sectional area (CSA) | Pre  Post | pQCT |
| Bio-Impedanz-Analysis (BIA) | Weekly | Tanita |
| Perceived pain (VAS) | Every Session | Questionaire |

#### References

Abdulla, A. J., Jones, P. W. & Pearce, V. R. (1999). Leg cramps in the elderly: prevalence, drug and disease associations. *International Journal of Clinical Practice, 53*(7), 494–496.

Miller, T. M., & Layzer, R. B. (2005). Muscle cramps. *Muscle Nerve*, *32*(October), 431–442.

Miller, K. C., & Knight, K. L. (2009). Electrical stimulation cramp threshold frequency correlates well with the occurence of skeletal muscle cramps. *Muscle Nerve*, *39*(March), 364–368.

Miller, K. C., Knight, K. L., Wilding, S. R., & Stone, M. B. (2012). Duration of Electrically Induced Muscle Cramp Increased by Increasing Stimulation Frequency. *Journal of Sport Rehabilitation*, *21*, 182–185.

Minetto, Marco Alessandro; Botter, Alberto; Ravenni, Roberta; Merletti, Roberto; De Grandis, D. (2008). Reliability of a novel neurostimulation method to study involuntary muscle phenomena. *Muscle Nerve*, *37*(January), 90–100.

Minetto, M. A., Holobar, A., Botter, A., & Farina, D. (2013). Origin and development of muscle cramps. *Exercise and sport sciences reviews*, *41*(1), 3–10.

Naylor, J. R. & Young, J. B. (1994). A general population survey of rest cramps. *Age and ageing*, *23*(5), 418–420.

Stone, M. B., Edwards, J. E., Babington, J. P., Ingersoll, C. D., & Palmieri, R. M. (2003). Reliability of an electrical method to induce muscle cramp. *Muscle Nerve*, (January), 122–123.

Stone, M. B., Edwards, J. E., Huxel, K. C., Cordova, M. L., Ingersoll, C. D., Babington, J. P., & Carolina, N. (2010). Threshold frequency of an electrically induced cramp increases following a repeated , localized fatiguing exercise. *Journal of Sports Sciences*, *28*(4), 399–405.

#### Listing of studies using EMS

The following two tables provide an overview of international studies that focused on adaptations to electrical muscle stimulation and cramp research.

**Table 2:** Listing of international studies that investigated adaptations to electromyostimulation

| Authors | Impulse type | Intensity | Impulse width [µs] | Frequency [Hz] | Duration of Stimulation [s] | Pause from stimulation [s] | Duty-cycle | Injuries | Fitnesslevel | Number of Training sessions | Weeks | Sessions per week |
| --- | --- | --- | --- | --- | --- | --- | --- | --- | --- | --- | --- | --- |
| Balogun et al. (1993) | monophasic 2-peak needleimpulse | - | 70 | 20 | 10 | 50 | 16,7 | No | Untrained | 18 | 6 | 3 |
| Balogun et al. (1993) | monophasic 2-peak needleimpulse | - | 70 | 45 | 10 | 50 | 16,7 | No | Untrained | 18 | 6 | 3 |
| Balogun et al. (1993) | monophasic 2-peak needleimpulse | - | 70 | 80 | 10 | 50 | 16,7 | No | Untrained | 18 | 6 | 3 |
| Currier und Mann (1983) | "Russian Current"-Sinusimpulse | 66,7% MVC ~ 45,8 mA | 450 | 50 | 15 | 50 | 23,1 | No | Untrained | 15 | 5 | 3 |
| Currier und Mann (1983) | "Russian Current"-Sinusimpulse | 88,4% MVC ~ 55,3 mA | 450 | 50 | 15 | 50 | 23,1 | No | Untrained | 15 | 5 | 3 |
| Eriksson et al. (1981) | Rectangular impulse | - | 500 | 200 | 15 | 15 | 50 | No | Trained students | 25 | 5 | 5 |
| Eriksson et al. (1981) | Rectangular impulse | - | 500 | 200 | 6 | 6 | 50 | No | Trained students | 25 | 5 | 5 |
| Fahey et al. (1985) | biphasic, asymetric rectangular Imulse | 45 mA | - | 50 | 10 | 5 | 66,7 | No | Untrained | 18 | 6 | 3 |
| Gondin et al. (2005) | biphasic, rectangular Imulse | 68 % MVC ~ 75 mA | 400 | 75 | 6,25 | 20 | 23,8 | No | Untrained | 32 | 8 | 3 |
| Gondin et al. (2005) | biphasic, rectangular Imulse | 68 % MVC ~ 75 mA | 400 | 75 | 4 | 20 | 16,7 | No | Untrained | 32 | 8 | 4 |
| Herrero et al. (2006) | biphasic, rectangular Imulse | 40 mA | 400 | 120 | 3 | 30 | 23,8 | No | Untrained | 8 | 4 | 2 |
| Herrero et al. (2006) | biphasic, rectangular Imulse | 66 mA | 400 | 120 | 3 | 30 | 9,1 | No | Untrained | 16 | 4 | 4 |
| Kubiak et al. (1987) | "Russian Current"-Sinusimpulse | 75% MVC | - | 50 | 15 | 50 | 23,1 | No | Untrained | 15 | 5 | 3 |
| Lai et al. (1988) | biphasic, asymetric Imulse | 64,60% | 200 | 50 | 5 | 5 | 50 | No | Untrained | 15 | 3 | 5 |
| Lai et al. (1988) | biphasic, asymetric Imulse | 52,00% | 200 | 50 | 5 | 5 | 50 | No | Untrained | 15 | 3 | 5 |
| Laughman et al. (1983) | "Russian Current"-Sinusimpulse | 33% MVC ~ 62,5 mA | - | 50 | 15 | 50 | 23,1 | No | Untrained | 25 | 5 | 5 |
| Maffiuletti et al. (2006) | biphasic, rectangular Imulse | 70% MVC ~ 64 mA | 400 | 75 | 6,25 | 20 |  | No | Untrained | 18 | 4 | 4,5 |
| Maffiuletti et al. (2002) | biphasic, rectangular Imulse | 60% MVC ~ 60 mA | 400 | 75 | 4 | 20 | 16,7 | No | Untrained | 16 | 4 | 4 |
| Mohr et al. (1985) | monophasic 2-peak needleimpulse | - | 45 | 50 | 10 | 10 | 50 | No | Untrained | 15 | 3 | 5 |
| Owens und Malone (1983) | "Russian Current"-Sinusimpulse | 60% MVC ~ 46,5 | 200 | 50 | 15 | 50 | 23,1 | No | Untrained | 10 | 1,5 | 6,7 |
| Owens und Malone (1983) | "Russian Current"-Sinusimpulse | 39% MVC ~ 34,6 | 200 | 50 | 15 | 50 | 23,1 | No | Untrained | 10 | 1,5 | 6,7 |
| Romero et al. (1982) | "Faraday"-Impulse (medium frequency) | - | - | 2000 | 4 | 4 | 50 | No | Untrained | 10 | 5 | 2 |
| Selkowitz (1985) | "Russian Current"-Sinusimpulse | 91% MVC ~ 59 mA | 450 | 50 | 10 | 120 | 7,7 | No | Trained students | 12 | 4 | 3 |
| Stefanovska und Vodovnik (1985) | monophasic Sinusimpulse | 5% MVC ~ 73,1 mA | 300 | 25 | 10 | 50 | 16,7 | No | Untrained | 24 | 4 | 6 |
| Stefanovska und Vodovnik (1985) | Monophasic rectangular impulse | 5% MVC ~ 43,12 mA | 300 | 25 | 10 | 50 | 16,7 | No | Untrained | 24 | 4 | 6 |

**Table 3:** Listing of international studies in the field of cramp research within a sport scientific context

| Author | Subjects [n] | Muscle | Stimulation type | Injuries |
| --- | --- | --- | --- | --- |
| Minetto et. al (2008) | 19 | M. abductor hallucis | Motor Point | No |
| Minetto et. al (2009) | 11 | M. abductor hallucis | Motor-Point | No |
| Minetto und Botter (2009) | 11 | M. abductor hallucis  M. flexor hallucis brevis  M. gastrocnemius | Motor Point | No |
| Minetto et. al (2009) | 15 | M. abductor hallucis | Motor Point | No |
| Bertolasi et. al (1993) | 10 | M. flexor hallucis brevis | Nerve | No |
| Braulick et. al (2012) | 9 | M. flexor hallucis brevis | Nerve | No |
| Caress et. al (2000) | 14 | M. abductor hallucis | Nerve | No |
| Miller und Knight (2007) | 23 | M. flexor hallucis brevis | Nerve | No |
| Miller und Knight (2009) | 31 | M. flexor hallucis brevis | Nerve | No |
| Miller et. al (2010) | 12 | M. flexor hallucis brevis | Nerve | No |
| Miller et. al (2012) | 20 | M. flexor hallucis brevis | Nerve | No |
| Miller et. al (2012) | 20 | M. flexor hallucis brevis | Nerve | No |
| Serrao et. al (2007) | 13 | M. flexor hallucis brevis | Nerve | No |
| Stone et. al (2003) | 16 | M. flexor hallucis brevis | Nerve | No |
| Stone et. al (2010) | 16 | M. flexor hallucis brevis | Nerve | No |
| Jung et. al (2005) | 13 | M. gastrocnemius | Voluntary | No |
| Khan und Burne (2007) | 13 | M. gastrocnemius | Voluntary | No |
| Roelefeld et. al (2000) | 8 | M. gastrocnemius | Voluntary | No |
